# Editorial: Nanobiophotonics and Related Novel Materials Aimed at Biosciences and Biomedicine

**DOI:** 10.3389/fbioe.2022.898752

**Published:** 2022-04-20

**Authors:** Carlo Altucci, Rajendra Kurapati, Eden Morales-Narváez

**Affiliations:** ^1^ Department of Advanced Biomedical Science, Università degli Studi di Napoli Federico II, Napoli, Italy; ^2^ Istituto Nazionale di Fisica Nucleare (INFN), Sec. of Napoli, Napoli, Italy; ^3^ School of Chemistry, Indian Institute of Science Education and Research Thiruvananthapuram, Thiruvananthapuram, India; ^4^ Biophotonic Nanosensors Laboratory, Centro de Investigaciones en Óptica A. C., León, Mexico

**Keywords:** 2D nanobiomaterials, biophotonics, optically active materials, biofunctionalization, nanomedicine

Nanobiophotonics is an emergent multidisciplinary field embracing nanotechnology, photonics, materials, and biomedicine. In terms of their use in nanobiophotonics, soft- and bio-nanomaterials offer a high level of structural diversity and tunability. As a matter of fact, nanostructures such as two-dimensional nanosheets and other nanoscale building blocks, exhibit exciting functions such as an enormous surface-to-volume ratio and unique optical, chemical, and electronic properties. These functions enhance the ability of metallic or semiconducting nanomaterials to absorb, reflect, and interact with light, which is of primary importance in developing nanophotonics, where photonics merges with nanoscience and nanotechnology. Moreover, these materials can easily be engineered and functionalized, thus modifying their organization at the nanoscale and consequently their physico-chemical properties. The advances in understanding the behavior of soft matter and biomaterials at a molecular level, with the help of photonics techniques, are being translated into functional materials systems and devices, which take advantage of newly discovered and specifically created morphologies with desired properties. In such scenarios, photonics and nano-biosciences are correlated. On the one hand, photonics provides a powerful set of tools with which to investigate nanobiosciences and related novel materials. On the other hand, the investigation of optical linear and nonlinear properties of these materials certainly widens the frontier of the most recent photonics achievements.

Such a multidisciplinary context—rapid advancement in so many directions from functionalized soft matter and novel biomaterials aimed at applications in biosciences and biomedicine to nanophotonics including nonlinear optics—finds its natural platform in the scientific communities at *Frontiers*, with its cross-sectoral approach to publication, for both undertaking and presenting this inter-disciplinary Research Topic. In this context, this article collection highlights recent advancements in biocompatibility, biosensing, photo-bio reactions, and photo-thermal effects, taking advantage of nanophotonic phenomena with (potential) applications in biosciences and biomedicine.

Nanomedicine, with issues such as biocompatibility and biodegradability of novel nanomaterials, is among the particular areas of research in scope for this Research Topic. The contribution from Mitev et al. explored the cytocompatibility of nanodiamonds purified with novel plasma- and microwave-based techniques. Thanks to the large number of tested technologies to tailor the surface characteristics of the realized nanodiamonds, the authors proved in cultures of human fibroblast cells that enhanced viability is obtained in the presence of many types of processed nanodiamonds, indicating the potential for dermal applications of these nanomaterials.

Yet, the application of novel hetero-hybrid structures, such as molecularly imprinted nanoparticle receptors, assembled with nanoplasmonic probes, is at the fundament of novel biosensing applications in Cennamo et al. Here the authors have developed soft, deformable, molecularly imprinted nanoparticles (nano-MIPs) in combination with nano-plasmonic sensor chips realized on polymeric substrates to realize highly sensitive bio/chemical nano-sensors (d_mean_ < 50 nm); these are capable of selectively binding Bovine Serum Albumin (BSA), which was used as the test agent. The special, deformable character of the nano-MIPs enabled the significant enhancement of the limit of detection of the plasmonic bio/sensor, allowing the detection of the low femtomolar concentration of analyte (∼3 fM), thus attaining ultralow detections, down to the quasi-single molecule.

A novel biosensing application focused on the detection of biologically relevant molecules such as albumin proteins is a key concept in a study by Vespini et al.; they have realized a novel type of biofunctionalized gold nanoparticle, based on pyroelectrohydrodynamic jet printing technology, to sense biomolecules by means of surface-enhanced Raman spectroscopy. Here, the signal behavior was also tested in the case of the BSA as a model analyte to demonstrate the affinity with biomolecules by means of strong SERS activity across the whole spot area of the probing laser beam.

Di Meo et al., developed a plasmonic metasurface, in particular a gold metasurface on a silicon chip, for the detection of deoxyribonucleic acid (DNA) fragments. The authors biofunctionalized this chip to accomplish a biosensing platform based on surface-enhanced infrared absorption spectroscopy exhibiting an analytical sensitivity in the femtomolar range. The team also discussed the advantages of this approach in terms of processing time, versatility, and implementation.

A molecular model system for photo-crosslinking between nucleic acids and proteins was analyzed by Bende et al., who discussed the mechanisms and molecular pathways required for such an important reaction to occur. In view of the results, it can be concluded that the DNA–protein crosslinking reaction can be induced by the external electromagnetic field *via* the dimerization reaction between the six-membered rings of the uracil–benzene pair at the electronic excited-state level of the complex. This can shed light on important biological photo-reactions, indicating that during the photo-process induced by a UV light pulse both scenarios can take place: a biologically positive internal conversion, serving as a protection mechanism against the UV radiation, and biologically negative dimerization leading to DNA damage.

Photonic 2D materials and their interactions with human cells, normal, and tumor type, have been discussed by Rusciano et al., where the photothermal effect of 2D-nanoflakes has been studied at a single-cell level, based on Raman microscopy bioimaging, mostly for the sake of anti-cancer application in theranostics. It has been demonstrated that irradiation of human breast cancer MCF7 cells targeted with MoS_2_ nanosheets causes a relevant photothermal effect, which is particularly high in the presence of MoS_2_ nanosheet aggregates. Laser-induced heating is strongly localized near such particles which, in turn, tend to accumulate near the cytoplasmic membrane. As an outcome of the experiment, novel indications for tuning the nanosheet fabrication process are traced with the aim of exciting biomedical applications such as anti-cancer drugs based on the photothermal action against tumor cells and specially designed drug delivery systems.

Exploring these new *Frontiers* of research and interdisciplinarity is a fascinating journey toward novel prospects. We can indeed foresee a tremendous impact for the next 10 years in terms of novel nanomaterials being properly biofunctionalized and treated through photonics techniques given the striking applications in nano-biotechnology, nano-photonics, and nanosciences in general. Our hope and our main scope with this Research Topic is to give valuable and useful insights into this exciting field.

